# Agreement between aggregate and individual-level measures of income and education: a comparison across three patient groups

**DOI:** 10.1186/1472-6963-11-69

**Published:** 2011-03-31

**Authors:** Carlo A Marra, Larry D Lynd, Stephanie S Harvard, Maja Grubisic

**Affiliations:** 1 Faculty of Pharmaceutical Sciences, University of British Columbia, Vancouver, BC, Canada; 2 Centre for Health Evaluation and Outcome Sciences, Providence Health Research Institute, Vancouver, BC, Canada; 3 School of Population and Public Health, University of British Columbia, Vancouver, BC, Canada

**Keywords:** Socio-economic status, income, education, aggregate-level, individual-level asthma, diabetes, rheumatoid arthritis

## Abstract

**Background:**

The association between lower socioeconomic status and poorer health outcomes has been observed using both individual-level and aggregate-level measures of income and education. While both are predictive of health outcomes, previous research indicates poor agreement between individual-level and aggregate-level measures. The purpose of this study was to determine the level of agreement between aggregate-level and individual-level measures of income and education among three distinct patient groups, specifically asthma, diabetes, and rheumatoid patients.

**Methods:**

Individual-level measures of annual household income and education were derived from three separate surveys conducted among patients with asthma (n = 359), diabetes (n = 281) and rheumatoid arthritis (n = 275). Aggregate-level measures of income and education were derived from the 2001 Canadian census, including both census tract-and dissemination area-level measures. Cross-tabulations of individual-level income by aggregate-level income were used to determine the percentage of income classifications in agreement. The kappa statistic (simple and weighted), Spearman's rank correlations, and intra-class correlation coefficient (ICC) were also calculated. Individual-level and aggregate-level education was compared using Chi-Square tests within patient groups. Point biserial correlation coefficients between individual-level and aggregate-level education were computed.

**Results:**

Individual-level income was poorly correlated with aggregate-level measures, which provided the worst estimations of income among patients in the lowest income category at the individual-level. Both aggregate-level measures were best at approximating individual-level income in patients with diabetes, in whom aggregate-level estimates were only significantly different from individual-level measures for patients in the lowest income category. Among asthma patients, the proportion of patients classified by aggregate-level measures as having a university degree was significantly lower than that classified by individual-level measures. Among diabetes and rheumatoid arthritis patients, differences between aggregate and individual-level measures of education were not significant.

**Conclusions:**

Agreement between individual-level and aggregate-level measures of socioeconomic status may depend on the patient group as well as patient income. Research is needed to characterize differences between patient groups and help guide the choice of measures of socioeconomic status.

## Background

Socioeconomic status (SES) has been shown to be associated with health outcomes among the general population [1-3] as well among various patient groups, including asthma [4-10], rheumatoid arthritis (RA) [11,12], and diabetes mellitus (DM) patients [13]. Health outcomes associated with SES include level of asthma control [4], hospital admissions [5,8], emergency department and physician visits [6,7,9], and asthma-related mortality among asthmatics, disease activity, physical and mental health, and quality of life among individuals with RA [11,14], and hospitalizations among diabetics [13].

The association between lower SES and poorer health outcomes has been observed using individual-level measures of SES as well aggregate-level measures, such as those available from census data. While SES measures at both of these levels may be predictive of health outcomes, the validity of using aggregate-level measures as a proxy for individual-level measures is debatable. In Canada, studies aimed at quantifying the relationship between individual-level SES measurements and aggregates derived from Canadian census data have generally indicated poor agreement. This finding is consistent across studies using individual-level measures derived from self-report [15], from structured interviews [16], and from public health insurance data [17,18]. These studies indicate that aggregate measures from the Canadian census function to mask variation in individual-level measures, the latter being more sensitive to poverty and poor health outcomes. Studies using US census data further suggest that aggregate-level SES measures reflect a construct distinct from individual-level ones [19,20].

Despite their limitations, aggregate-level measures are considered appropriate for use when individual-level data are lacking [21]. Particularly in research using administrative data, aggregate measures are often the only available means to adjust for SES. In this context, the question remains whether aggregate-level measures perform equally well as individual-level proxies across different patient groups or whether the discrepancy between aggregate and individual-level measures is exaggerated in some populations. The question also remains whether there are differences across patient groups according to the aggregate-level measure used.

In Canada, the smallest geographical area for which all census variables are available is the dissemination area (DA), which typically contains 400 to 700 residents [22]. Studies may also utilize data from larger census units, such as the census tract (CT), containing 2500 to 8000 residents [23]. While all of Canada is divided into DAs, only regions with a population of 50,000 or more are divided into CTs, which may lead to differences between DA-and CT-level data.

The purpose of this study was to determine the level of agreement between aggregate-level and individual-level measures of income and education among three distinct patient groups, specifically asthma, DM, and RA patients.

## Methods

### Data

Individual-level measures of annual household income and education were derived from three separate self-report surveys conducted among patients with asthma (n = 359), DM (n = 281) and RA (n = 275), respectively. The methods for these surveys have been published previously [14,24,25]. All patients were recruited from British Columbia, Canada, and the samples are considered to be representative of the English-speaking, adult members of these patient groups in this region. Ethical approval for all three studies was obtained from the University of British Columbia.

Patients with asthma completed the surveys in years 2000 and 2005, patients with RA in 2002, and patients with DM in 2008. All patients completed the survey independently. All surveys included the same items on income and education, which pertained to annual household income prior to any deductions and to certificates, degrees, or diplomas obtained. All surveys collected patients' age, sex, and residential postal codes. Patients whose residential postal codes were missing were excluded from the study.

Aggregate-level measures of income and education were derived from the 2001 Canadian census data, available online from the University of British Columbia. Aggregate-level income was based on the median household income for the census level, which is the self-reported annual household income prior to any deductions [26]. Education was evaluated by highest level of schooling, which in the Canadian census refers to the self-reported "highest grade or year of elementary or secondary (high) school attended, or to the highest year of university or college education completed". Patients with the census classification 'university, with university degree' were categorized as having a university degree and all other patients were categorized as not having a university degree [26].

### Statistical Methods

Demographic variables between the three patient groups were compared using ANOVA for continuous variables and Pearson's Chi-Square and Fisher's exact tests for categorical variables, where appropriate. Statistics Canada's Postal Code Conversion File was used to link patients' postal codes to their corresponding CT and DA [27]. The inflation factor from the Canadian Consumer Price Index was used to adjust individual-level incomes from across survey years to their 2001 income equivalents [28]. Both individual-level and aggregate-level income were categorized as less than $20,000, between $20,000 and $50,000, and greater than $50,000 [24]. To determine the agreement between aggregate-level and individual-level income, 3 × 3 cross-tabulations of individual-level income category by CT-level income category and of individual-level income category by DA-level income category were produced. This output what used to determine the percentage of cases for which individual-level and DA/CT-level income categories were in agreement. The kappa statistic (simple and weighted) and Spearman's rank correlations were calculated to determine the degree of nonrandom agreement between individual-level and DA/CT-level income. The intra-class correlation coefficient (ICC) was also calculated using the 2-way mixed model for absolute agreement [29]. For the ICCs, levels of agreement were adopted as proposed by Fleiss, i.e., <0.40 poor, 0.40-0.75 fair to good, and ≥0.75 excellent [30].

Both individual-level and aggregate-level education were categorized as at least a university degree or less than university degree. In the analysis of aggregate-level education, only the census population in age groups 20-24 and higher was included. Individual-level and aggregate-level education was compared using Chi-Square tests within patient groups. Point biserial correlation coefficients between individual-level and DA/CT level education were also computed.

## Results

### Patient groups and Number of Corresponding CTs and DAs

Patients in the asthma sample (n = 359) resided in 198 discrete CTs and 321 discrete DAs, representing a total of 1,552,655 and 188,235 census respondents, respectively. Patients in the DM sample (n = 281) resided in 169 discrete CTs and 261 discrete DAs, representing a total of 1,166,395 and 163,095 census respondents, respectively. Patients in the RA sample (n = 276) belonged to 144 discrete CTs and 226 discrete DAs, representing a total of 833,735 and 157,500 census respondents, respectively.

### Individual and Aggregate-level Patient Sociodemographics

Table 1 shows the sociodemographic characteristics of each of the three patient groups. Patients in the asthma sample were significantly younger than those in the RA and DM samples (p < 0.0001) and there was a significantly greater proportion of females in the RA sample (p < 0.0001). Individual-level household income was highest among patients with DM, followed by patients with asthma and RA, respectively (p < 0.0001, with all pairwise comparisons p < 0.0001). There were no significant differences in the proportions of patients classed by CT or DA as having a university degree between the patient groups. The proportion of RA patients reporting a university degree was significantly lower than both the proportions of asthma patients (p < 0.0001) and DM patients (p < 0.0001). The difference between the asthma and diabetes samples in individual-level university education was not significant (p = 0.21).

**Table 1:** Characteristics of the study participants

	Asthma n = 359	Rheumatoid Arthritis n = 276	Diabetes n = 281	p-value*
**Characteristic**	**Mean (sd†) or n (%)**	**Mean (sd) or n (%)**	**Mean (sd) or n (%)**	

Age (years)	36.8 (8.4)	61.2 (13.8)^a^	56.9 (13.1)	<0.0001

Sex (males)	128 (35.7)	58 (21.2)^b^	147 (52.3)	<0.0001

Self-reported Income				
<20,000	90 (29.8)	44 (19.5)	34 (14.6)	
20,000-50,000	84 (27.8)	99 (43.8)	76 (32.6)	
>50,000	128 (42.4)	83 (36.7)	123 (52.8)	<0.0001

DA‡-Household Income				
<20,000	24 (6.7)	8 (3.0)	7 (2.5)	
20,000-50,000	180 (50.4)	128 (47.8)	112 (40.1)	
>50,000	153 (42.9)	132 (49.3)	160 (57.4)	0.001

CT§- Household Income				
<20,000	10 (2.8)	0	4 (1.5)	
20,000-50,000	192 (53.8)	76 (38.0)	86 (32.2)	
>50,000	155 (43.4)	124 (62.0)	177 (66.3)	<0.0001

Individual-level University degree	124 (34.5)	45 (17.3)^c^	82 (29.8)^d^	<0.0001

Aggregate-level University Degree (expected)-DA	91 (25.3)	50 (18.1)	71 (25.2)	0.06

Aggregate-level University Degree (expected)- CT	93 (25.9)	59 (21.3)	74 (26.4)	0.39

^a^2 missing; ^b^16 missing; ^c^6 missing; ^d^6 missing;*all p-values were obtained using Chi-square test except for age where ANOVA was used. †Standard deviation; ‡ Dissemination Area; § Census Tract

### Agreement: Individual-level and DA/CT Income Measures

In all patient groups, the proportion of patients who reported incomes under $20,000 per year was significantly higher than the proportion of patients classed in this income category by DA or CT (all p-values <0.0001). In the asthma group, the proportion of patients who reported an income between $20,000 and $50,000 per year (27.8%) was also significantly lower than the proportions of patients classed in this income category by DA (50.4%; p < 0.0001) or by CT (53.8%; p < 0.0001); the proportions in the highest income category (>$50,000 per year) were similar. In the RA group, the proportion of patients reporting an income over $50,000 per year was significantly lower than the proportions of patients classed in this income category by DA (p = 0.005) or by CT (p < 0.0001). Among DM patients, there were no significant differences between the proportions of patients reporting incomes between $20,000 and $50,000 or >$50,000 per year and the proportions classed in these categories by DA and CT, respectively.

The Spearman's rank correlations, weighted kappa coefficients, and intra-class correlations indicating the association between individual-level and CT-level income measures, and individual-level and DA-level income measures, are shown in Table 2. Following the designations proposed by Fleiss (<0.40 poor, 0.40-0.75 fair to good, and ≥0.75 excellent), the ICCs generally indicated poor agreement between individual-level and aggregate-level income measures among all patient groups.

**Table 2:** Spearman's rank correlation, intra-class correlation and weighted kappa coefficients for the association of area-based and self-reported household incomes

	Census tract			Dissemination area		
	
	r_s_	ICC (95%CI)	k (95%CI)	r_s_	ICC (95%CI)	k (95%CI)
**Asthma**	0.28	0.25 (0.13,0.35)	0.20 (0.13,0.27)	0.35	0.29 (0.17,0.39)	0.24 (0.16,0.32)
**Rheumatoid Arthritis**	0.23	0.13(-0.01,0.27)	0.13(0.02,0.23)	0.29	0.15 (0.03,0.28)	0.16(0.06,0.25)
**Diabetes**	0.35	0.26 (0.13,0.38)	0.27 (0.17,0.37)	0.33	0.27 (0.15,0.38)	0.23 (0.13,0.33)

r_s _= Spearman's rank correlation

ICC(3,1) = Intra-class correlation coefficient (2-way mixed model, absolute agreement)

k = Weighted kappa coefficient

The extent of perfect agreement between individual-level and DA-level and CT-level groupings of income is illustrated in Tables 4 and 5, respectively. Among all patient groups, both for CT-level and DA-level income measures, agreement with individual-level measures was less frequent within the lowest income grouping. Of note, of the nearly 20% of RA patients who reported an income under $20,000 in the survey, none were correctly classed in this income grouping by DA or CT (Tables 4 and 5). Overall, across all patient groups there were no significant differences between DA-level and CT-level income groupings in the proportion of cases for which there was perfect agreement with individual-level measures (Table 3).

**Table 3:** Percentage of cases in perfect agreement between individual-level and DA/CT income groupings

	Asthma n(%)	Diabetes n(%)	Rheumatoid Arthritis n(%)	p-value*
DA	141 (47)	128(55)	109 (49)	0.17
CT	135 (45)	137 (62)	80 (49)	0.0005
p-value*	0.62	0.14	0.99	

*Chi-square test

**Table 4:** Percentage of cases in perfect agreement between individual-level and DA income groupings

	Asthma n(%)	Diabetes n(%)	Rheumatoid Arthritis n(%)	p-value*
<20,000	12 (13)	2 (6)	0 (0)	0.03**
20,000-50,000	52 (63)	37 (49)	56 (58)	0.23
>50,000	77(60)	89 (72)	53 (64)	0.12

*Chi-square test; **Fisher's exact test

**Table 5:** Percentage of cases in perfect agreement between individual-level and CT income groupings

	Asthma n(%)	Diabetes n(%)	Rheumatoid Arthritis n(%)	p-value*
<20,000	5 (6)	2 (7)	0 (0)	0.44**
20,000-50,000	54 (64)	36 (49)	31 (44)	0.03
>50,000	76 (60)	99 (83)	49 (74)	0.0002

*Chi-square test; **Fisher's exact test

### Agreement: Individual and Aggregate-level Education Measures

Table 6 shows the comparison of individual-level university education to DA-and CT-level measures of university education. In patients with asthma, CT-and DA-level census data indicated nearly equal proportions of patients with a university degree. However, this proportion was significantly lower than the proportion of asthma patients who reported having a university degree in the survey (35%) (p = 0.01). For the RA and DM patient groups, differences between individual-level and aggregate-level measures of education, respectively, were not significant.

**Table 6:** Percentage of patients with a university degree by individual- and aggregate-level measures

	Individual-level University Degree n(%)	CT-level University Degree (expected) n(%)	p-value*	DA-level University Degree (expected) n(%)	p-value*
Asthma	124 (34.5)	93 (25.9)	0.01	91 (25.3)	0.01
Diabetes	82 (29.8)	74 (26.4)	0.40	71 (25.2)	0.25
Rheumatoid Arthritis	45 (17.3)	59 (21.3)	0.26	50 (18.1)	0.85

*Chi-square test

Point biserial correlations between individual-level university degree and CT-level measures of education (i.e., proportion of the population with a university degree) were weak within all patient groups (asthma = 0.31; DM = 0.18; RA = 0.28). Compared to CT-level measures, DA-level measures of education were not more highly correlated with individual-level measures (asthma = 0.28; DM = 0.12; RA = 0.25).

## Discussion

This study is the first to compare the agreement between individual-level and aggregate-level measures of income and education among three distinct patient groups. The results suggest that the ability of aggregate-level measures to approximate individual-level measures of SES may vary by the patient group as well as patient income.

In this study, individual-level income was poorly correlated with CT-and DA-level measures, which is consistent with several other reports using Canadian census data [16,17,31,32]. Our findings are also similar to those of Southern and colleagues [31] who observed that census-level measures provided the worst estimations of income among lower-income households. In all three patient groups, significantly more patients reported being in the lowest income category than were classed as such by either aggregate-level measure. Among asthma patients, this discrepancy reflects CT-and DA-level measures having classed lowest-income patients in the middle income category, while among RA patients it points to aggregate-level measures having classed middle-income patients in the highest income category. Thus, aggregate measures of income tended to classify patients in higher income categories relative to individual-level measures. Notably, among RA patients who in the survey reported being in the lowest income category, individual-level incomes were never in agreement with the corresponding DA-or CT-level measures.

Both CT-and DA-level measures were best at approximating individual-level income in patients with DM, in whom aggregate-level values were only significantly different from individual-level values for patients in the lowest income category. The frequency of perfect agreement between aggregate-level and individual-level measures of income was also highest overall among DM patients, the only patient group in which both CT-and DA-level values agreed with Individual-level values in more than fifty percent of cases. As well as performing best among DM patients, both CT and DA-level measures best approximated individual-level measures of income among all patients in the highest income categories. Accordingly, across patient groups and income categories, the greatest proportion of cases in perfect agreement with aggregate-level measures was observed among DM patients of the highest income category.

With respect to education, all point biserial correlations between individual-level and aggregate-level measures were weak, with no differences between CT-and DA-level measures. Despite the weak correlations across all patient groups, among RA and DM patients there were no significant differences between individual-level and aggregate-level measures in the proportions of patients classified as having a university degree. The only difference in these proportions was observed within the asthma group, where the proportion of patients who reported having a university degree in the survey was significantly higher than the proportions in the corresponding CT-and DA-level populations.

These findings should be taken in context with the limitations of the study. First, individual-level income and education among our three patient groups was self-reported and could not be verified, and thus reporting bias may have affected the SES measures that were derived from surveys. However, the same can be said of census measures, which are also self-reported; in Canada, census measures are the most accessible population-based data and no 'objective' measures of income and education are available for the Canadian population. It should also be noted that, despite the risk of bias, self-reported measures of SES remain powerful predictors of health outcomes [33]. Ultimately, the absolute accuracy of the individual-level measures does not affect the conclusions regarding their agreement with aggregate-level measures.

In this study, individual-level measures are assumed to be better than aggregate-level measures, an assumption that follows from evidence that individual-level measures are more strongly associated with health outcomes [19]. However, this assumption could be inaccurate under some circumstances. It is possible that among some patients, income reported in a cross-sectional survey is not representative of prior income, e.g., income before retirement among older patients or income prior to disease onset among patients with work disability. This could explain the pattern of non-agreement observed here among RA patients with the lowest individual-level incomes, as RA patients are known to have a high burden of work disability [34,35]. In these cases, aggregate-level measures could reflect a prior income sustained over a longer period and therefore be more representative of SES. In addition, individual-level education may not be a good measure of SES among some patients, such as women among the oldest old, whose husband's educational attainment may be a better measure of SES than their own [36]. In this context, aggregate-level measures of education, which reflect contextual effects, may be more representative of an individual's SES. Finally, the aggregate-level data used here is from 2001, while individual-level data was collected in 2000, 2002, 2005 and 2008. Although methods were employed to correct income for inflation, no adjustment could be made to address the time lapse between the collection of individual-level measures and this could be a source of bias. However, given the mean age of the survey participants in the three patient groups, income and education status may be expected to have been relatively stable across the study periods and comparable to 2001 census measures.

## Conclusions

This study shows that the agreement between individual-level and aggregate-level measures of SES may depend on the patient group as well as patient income. While research is needed to characterize patterns of differences between patient groups to help guide the choice of SES indicators, the use of both individual-level and aggregate-level measures is advised in studies of health outcomes.

## Competing interests

The authors declare that they have no competing interests.

## Authors' contributions

CAM was involved in the conception of the study, participated in the study design and data interpretation and was involved in revising the manuscript critically for important intellectual content and approving the final version. LDL was involved in the conception of the study, participated in the study design and data interpretation and was involved in revising the manuscript critically for important intellectual content and approving the final version. SSH was involved in data interpretation, drafted the manuscript and gave final approval for the version to be published. MG performed the statistical analysis, interpreted the data and revised the manuscript critically for important intellectual content.

## Authors' information

CAM is a Government of Canada Research Chair in Pharmaceutical Outcomes and a Michael Smith Foundation for Health Research Scholar in Health Services Research. LDL is a Canadian Institutes of Health Research New Investigator and a Michael Smith Foundation for Health Research Scholar in Population Health. Both CAM and LDL are Associate Professors in the Faculty of Pharmaceutical Sciences at the University of British Columbia. SSH is a researcher in the Faculty of Medicine at the University of British Columbia. MG is a biostatistician in the Faculty of Pharmaceutical Sciences at the University of British Columbia.

## Pre-publication history

The pre-publication history for this paper can be accessed here:

http://www.biomedcentral.com/1472-6963/11/69/prepub
